# The fate of patients after failed epicardial ablation of atrial fibrillation

**DOI:** 10.1186/s13019-021-01635-3

**Published:** 2021-09-06

**Authors:** Giuseppe Nasso, Roberto Lorusso, Arash Motekallemi, Angelo M. Dell’Aquila, Nicola Di Bari, Ignazio Condello, Marco Moscarelli, Saverio Iacopino, Giuseppe F. Serraino, Pasquale Mastroroberto, Giuseppe Santarpino, Giuseppe Speziale

**Affiliations:** 1 Department of Cardiac Surgery, Anthea Hospital, GVM Care and Research, Via Camillo Rosalba, 35/38, Bari, Italy; 2 Cardio-Thoracic Surgery Department, Heart and Vascular Centre, Maastricht University Medical Centre (MUMC), Maastricht, Netherlands; 3 Cardiovascular Research Institute Maastricht (CARIM), Maastricht, Netherlands; 4 Department of Cardiac Surgery, Münster Universität, Münster, Germany; 5 Department of Cardiac Surgery, “Aldo Moro” University, Bari, Italy; 6 Department of Experimental and Clinical Medicine, Magna Graecia University, Catanzaro, Italy; 7 Department of Electrophysiology, Maria Cecilia Hospital, GVM Care and Research, Cotignola, RA Italy; 8 Department of Cardiac Surgery, Paracelsus Medical University, Nuremberg, Germany

**Keywords:** Atrial fibrillation, Surgical ablation of atrial fibrillation, Catheter ablation of atrial fibrillation

## Abstract

**Background:**

Much debate is still going on about the best ablation strategy—via endocardial or epicardial approach—in patients with atrial fibrillation (AF), and evidence gaps exist in current guidelines in this area. More specifically, there are no clear long-term outcome data after failed surgical AF ablation.

**Methods:**

Since June 2008, 549 surgical AF ablation procedures through a right minithoracotomy were performed at our institution. From 2008 to 2011, a unipolar radiofrequency device was used (151 patients), whereas from 2011 to 2020 a bipolar radiofrequency device was used (398 patients). Patients were scheduled for surgery on the basis of the following criteria: recurrent episodes of paroxysmal or persistent lone AF refractory to maximally tolerated antiarrhythmic drug dosing and at least one failed cardioversion attempt. Besides the recommended follow-up by the local cardiologist, starting from 2021, surviving patients were asked to undergo assessment of left ventricular function and to complete a questionnaire addressing quality of life and predisposing factors for recurrent AF.

**Results:**

At a mean follow-up of 77 months, the rate of AF recurrence was 20.7% (n = 114). On multivariate analysis, impaired left ventricular ejection fraction (58 patients, 51%, *p* = 0.002), worsening of European Heart Rhythm Association (EHRA) symptom class (37 patients, 32%, *p* = 0.003) and cognitive decline or depression (23 patients, 20%, *p* = 0.023) during follow-up were found to be significantly associated with AF recurrence.

**Conclusions:**

Surgical AF ablation through a right minithoracotomy is safe, but a better outcome could be achieved using a hybrid approach. Patients after initial failed surgical AF ablation show worsening of cardiac function, clinical status and quality of life at follow-up compared to patients with successful AF ablation.

## Background

Much debate is still going on about the best ablation strategy for the treatment of atrial fibrillation (AF), which prompted the latest 2020 update of the European Society of Cardiology/European Association of Cardio-Thoracic Surgery (ESC/EACTS) guidelines for the management of AF for improving adherence to practice guidelines among healthcare professionals [1]. Among the 2020 ESC/EACTS recommendations, cardiac surgery may play a role in specific patient subsets. On one hand, catheter ablation may be considered as an initial therapy in selected patients who remain highly symptomatic despite drug therapy or as an alternative in AF patients refractory to antiarrhythmic drug treatment. On the other hand, however, the relevance of achieving complete electrical isolation of the pulmonary veins (PVs) is strongly underscored. Durable PV isolation with catheter ablation is difficult to achieve, with PV reconnection rates as high as 70% [1].

However, despite the large number of catheter ablation procedures performed, only few patients undergo multidisciplinary heart team discussion for proper decision making about patient-tailored or optimal therapeutic strategy, like hybrid AF ablation procedure [1].

In particular, by comparing guideline recommendations of 2016 vs 2020, catheter or surgical ablation should be considered in patients with symptomatic persistent or long-standing persistent AF (Class IIb in 2016), but only AF catheter ablation for PV isolation is recommended for rhythm control after one failed or intolerant drug therapy (Class I in 2020) [1]. Thoracoscopic or hybrid surgical ablation for patients refractory to drug therapy or after failed percutaneous AF ablation stays as a Class IIa recommendation [1].

Unsuccessful PV isolation results in recurrent or persistent AF and may be even associated with worse outcome. It is well known that patients undergoing catheter ablation have an increased long-term risk for heart failure [2], regardless of left ventricular ejection fraction before the procedure [3]. At present, no data are available regarding patients with lone AF undergoing isolated surgical ablation by PV isolation [4].

### Aim of the study

The aim of this study, therefore, was to evaluate the long-term outcome of patients undergoing isolated surgical AF ablation, particularly in the patient subset with unavailable data on long-term success of cardioversion by evaluating clinical and echocardiographic status during follow-up.

## Methods

From June 2008 to December 2020, 549 surgical AF ablation procedures through a right minithoracotomy were performed at our institution (Gruppo Villa Maria (GVM) Care&Research, Anthea Hospital, Bari, Italy). Of these, 340 females (62%), 209 males (38%) did not show secondary causes of AF (e.g. mitral valve disease). From 2008 to 2011, a unipolar radiofrequency device was used (Estech, Cobra Adhere XL), whereas from 2011 to 2020 a bipolar radiofrequency device was used, i.e. a unidirectional device with two electrodes (Estech COBRA Fusion™ 150 Surgical Ablation System).

Part of our population and experience with isolated treatment of AF via right mini-thoracotomy has been described previously [5, 6]. The sample size is larger than that of the previous publications, which included patients who underwent surgical ablation of AF until September 2015. Patients were scheduled for surgery on the basis of the following criteria (which did not change over time): recurrent episodes of paroxysmal or persistent lone AF refractory to maximal tolerated doses of class IC or III antiarrhythmic agents, alone or in combination, and at least one failed electrical or pharmacological cardioversion attempt during the 6 months preceding surgical evaluation. Patients considered for surgery were suitable candidates for both percutaneous and surgical approach; they were informed about both procedures and the final decision was left to the patient’s discretion.

AF definition followed current guideline classification [1]. In particular, paroxysmal AF was defined as a self-terminating AF episode (up to 7 days), and persistent AF was defined as an AF episode lasting longer than 7 days, or requiring termination by electrical or pharmacological cardioversion [1].

Our operative technique has been described elsewhere [4, 6]. Briefly, a 3 to 4 cm right minithoracotomy was performed at the level of the 3rd intercostal space (Appendix 1—Fig. 2). The devices (Estech, Cobra Adhere XL and Estech COBRA Fusion™ 150) deliver bipolar or unipolar energy with the aim to obtain electrical isolation of the PV by temperature-controlled radiofrequency ablation of the atrial myocardium. All procedures were performed off-pump. A circular box lesion was created (Appendix 2—Fig. 3).

The right minithoracotomy approach was chosen for the easy access to the pulmonary vein box through the transverse and oblique sinus; although it implies the limitation of difficult access to the left auricle.

Patients were then referred to the local cardiologist with recommended follow-up at 6, 9, 12 months and then every 6 or 12 months depending on rhythm stability. In addition, starting from 2021, surviving patients were asked to undergo assessment of left ventricular function and to complete a questionnaire addressing quality of life and predisposing factors for recurrent AF, including alcohol and caffeine intake, smoking, and excess weight or weight loss. In addition, patients with diabetes, hypertension or obstructive sleep apnea requiring continuous positive airway pressure were asked if they were receiving appropriate treatment for their disease and were facing any difficulties in compliance and achieving optimal medical therapy.

Patients were divided into two groups based on whether they experienced a recurrence of electrocardiographic (ECG) or clinical AF by excluding the first 3 months post-procedure. The two groups were compared as for risk/triggering factors, and clinical and echocardiographic parameters recorded at last follow-up on the basis of patient-reported outcomes and questionnaire assessment on behavioral changes to reduce triggering factors (i.e. weight excess or loss, reduction or elimination of alcohol and caffeine intake, optimal management of diabetes or hypertension or sleep apneas). *Q*uality of life was “indirectly” evaluated and compared between groups using New York Heart Association (NYHA) functional classification and the European Heart Rhythm Association (EHRA) score of AF-related symptoms, as previously described [6].

In January 2021, the GVM Care&Research ethics board approved the retrospective study and, due to the characteristics of the analysis, the need for informed consent was waived.

### Statistical analysis

Data analysis was performed using Excel 2016 (Microsoft, Redmond, WA, USA) and statistical analysis using SPSS (IBM SPSS Statistics for Windows, Version 27.0. Armonk, NY, IBM Corp). Categorical variables are given as counts and percentages. Event-free estimate such as AF recurrence was determined using the Kaplan–Meier method. Possible risk factors for recurrent AF are reported in Table 1 and were used for the determination of the predictive model. To this purpose, an univariate analysis was performed first. Variables with a *p* value of 0.2 were included in a multivariable Cox regression model with stepwise selection to determine the independent predictors of AF recurrence. A *p* value of < 0.05 was considered statistically significant.

**Table 1:** Cox regression analysis for atrial fibrillation recurrence

	Patients with SR at follow up	Patients with failed ablation	Total/mean	Univariate	Multivariate	Hazard Ratio	95% confidence interval		
				%/ ± SD	*p* value	*p* value		Upper	Lower
Age, mean (SD)	63 ± 10	63 ± 10	63.08	± 9.85	0.44				
Type of ablation (bipolar)	314 (72%)	84 (73.7%)	398	72.5	0.857				
Complications, intraop	4 (0.9%)	3 (2.6%)	7	1.15	0.118	0.037	0.208	0,048	0,907
Complications, postop	3 (0.7%)	8 (7%)	11	1.81	0.64				
AF Rhythm (paroxysmal)	209 (48%)	76 (66.7%)	285	46.80	0.527				
AF Rhythm at discharge	20 (4.6%)	10 (8.8%)	30	4.93	0.781				
*Follow-up*									
Atrial fibrillation at follow-up	0	114	114	20.8	n.a				
Ejection fraction, mean (SD)	54 ± 6	55 ± 6	54.54	± 5.749	0.486				
Ejection fraction at follow-up, mean (SD)	54 ± 6	45 ± 11	52.38	± 8.153	0.000	0.000	0.942	0.920	0.964
NYHA class	1	1 ± 1	1.11	± 0.351	0.000	n.s	–		
Gained 10 kg	17 (3.9%)	9 (7.9%)	26	4.7	0.21				
Continued smoking	25 (5.7%)	20 (17.5%)	45	8.2	0.044	n.s	–		
Continued drinking	12 (2.7%)	15 (13.1%)	27	4.9	0.000	n.s	–		
CPAP at follow-up	20 (4.6%)	11 (9.6%)	31	7.6	0.022	n.s	–		
Hypertension at follow-up	124 (28%)	49 (43%)	173	33.7	0.241				
Diabetes at follow-up	25 (5.7%)	11 (9.6%)	36	6.6	0.858				
Caffeine consumption	16 (3.7%)	28 (24.6%)	44	7.22	0.000	n.s	–		
EHRA score mean (SD)	1	1 ± 1	1.08	± 0.292	0.065	0.008	0.192	0.057	0.644
Hospitalization for cardiac causes	9 (2%)	33 (28.9%)	42	6.90	0.000	n.s	–		
Cerebrovascular event at follow-up	20 (4.6%)	8 (7%)	28	4.60	0.017	n.s	–		
Cognitive impairment or depression	4 (0.9%)	36 (31.6%)	40	6.57	0.000	0.010	1.746	1.143	2.668

## Results

**Fig. 1:**
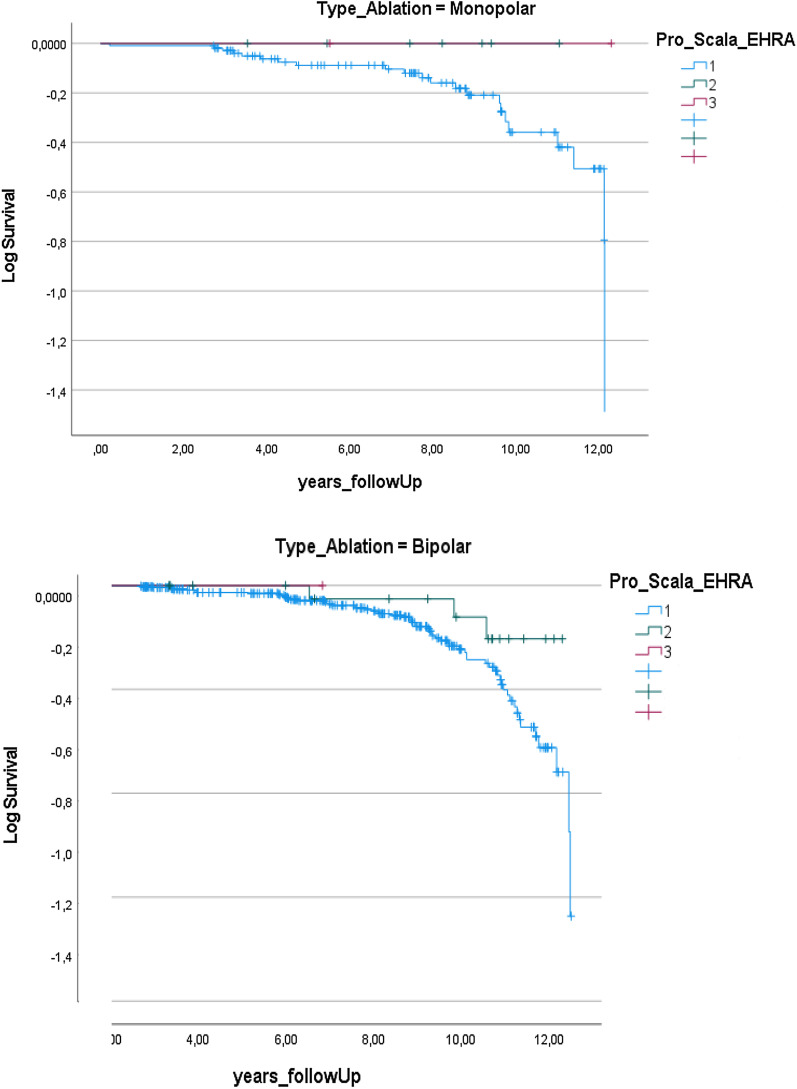
Patients undergoing unipolar (top of graph) or bipolar (bottom of graph) ablation. Blue curve: patients in EHRA class 1; Green curve: patients in EHRA class 2; Red curve: patients in EHRA class 3

All patients underwent surgical ablation due to paroxysmal AF in 264 (48.1%) patients and persistent AF in 285 (51.9%) patients. Mean age was 63 years (range 27–87 years). The AF ablation procedure was performed using the bipolar radiofrequency device in 398 (72.5%) patients and the unipolar radiofrequency device in 151 (27.5%) patients. No differences were observed between groups in the EHRA score at follow-up (Fig. 1). Intraoperative and postoperative complications were recorded in 1% (n = 6) of patients of both groups. The 30-day mortality was 0%. At discharge, 94.5% (n = 519) were in sinus rhythm and 5.5% (n = 30) were in AF. No patient required pacemaker implantation. At a mean follow-up of 77 months (range 2–152 months), 114 (20.8%) patients experienced AF recurrence. On univariate analysis, intraoperative complications, reduced left ventricular ejection fraction, higher NYHA class, continued alcohol and caffeine consumption, smoking, suboptimal management of continuous positive air pressure, hospitalization due to cardiac reasons and the occurrence of cerebrovascular events during follow-up were found to be associated with recurrent AF during follow-up (Table 1). On multivariate analysis, impaired left ventricular ejection fraction (*p* = 0.002), worsening of EHRA symptom class (*p* = 0.003) and cognitive decline or depression (*p* = 0.023) during follow-up were found to be significantly associated with AF recurrence (Table 1).

## Discussion

In our series of 549 patients undergoing isolated surgical AF ablation, at an average time of more than 6 years from the procedure about 1 in 5 patients developed AF recurrence. In multivariate analysis, impaired left ventricular ejection fraction, worsening of the EHRA symptom class and cognitive decline or depression during follow-up were found to be associated with AF recurrence. In other words, it is necessary to look for alternative/integrative strategies to reduce the risk for AF recurrence, given that this event negatively correlates with cardiac function, clinical status and quality of life of patients experiencing failed ablation procedures.

Monitoring of patients undergoing surgical or catheter ablation of AF has evolved remarkably over the last years. Current guidelines emphasize the relevance of patient-reported outcomes, meaning that if the patient feels better, the ablation procedure has been successful [1]. The issue of patient-reported outcomes has fostered a debate on the follow-up protocols to be adopted in ablated patients. There is general consensus that the risk of misdiagnosed AF, atrial high-rate episodes/subclinical AF, the need for Holter monitoring and loop recorders, and the use of smartphone apps and telemedicine, all seem to play a secondary role compared to the health status that comes directly from the patient. If symptoms related to AF assessed by the EHRA score have improved, successful ablation has been achieved [1].

Current guidelines also suggest that catheter ablation should be reserved for patients with AF which remains symptomatic despite optimal medical therapy [1]. Besides the clear indication for the need of providing practitioners and institutions with tools to measure the quality of care that AF patients receive so as to identify opportunities for improvement, the impact of lesion sets in addition to PV isolation is still uncertain.

### Safety

A first consideration based on the results of our study is that the ablation procedure can be performed safely through a right minithoracotomy. Theoretically, to achieve complete closure of the box lesion, 7 out of 10 patients that have undergone catheter ablation will need surgery to complete the hybrid procedure [1]. Obviously, this does not occur in clinical practice due to safety concerns surrounding surgical AF ablation, which is burdened by higher rates of periprocedural complications compared to catheter ablation [7]. However, prospective registry-based data show that approximately 4–14% of patients undergoing AF catheter ablation experience complications, 2–3% of which are potentially life-threatening. These complications occur mostly within the first 24 h after the procedure, but some may develop 1–2 months after ablation (like pulmonary vein stenosis). Periprocedural death is rare (< 0.2%) and usually related to cardiac tamponade. Indeed, these data derived from the current guidelines do not differ from those reported with thoracoscopic surgical ablation [1]. The use of a thoracoscopic approach is associated with a higher risk of pneumothorax and a low risk of cardiac tamponade, though similar to catheter ablation. Such complications, which have been recorded in 1% of our study population, can be safely managed through a right minithoracotomy performed under direct vision. This treatment option, which has been used in our center for the past 10 years, is not considered in the current guidelines.

Minimally invasive surgical ablation through a right minithoracotomy can also allow to address technical challenges when performing additional lesion lines (e.g. adjunctive Bachmann’s bundle ablation [8]), which seem to confer encouraging results but are not considered yet in current guidelines due to the lack of sufficient evidence*.* During our more than 10-year experience on 549 patients, in-hospital mortality was 0% and intraoperative and postoperative complications occurred in 2% of patients (n = 12) and were successfully treated in all of them*.* Such a complication rate is similar to that reported after catheter ablation. Therefore, in our opinion, performing a procedure that is accompanied by potential complications with risk of not achieving completeness of ablation lines may be questionable. The relevance of the hybrid approach for overcoming the limitations of catheter ablation and ensuring complete PV isolation should be considered by patients and healthcare professionals in their decision-making. Moreover, as supported by current guidelines, complete electrical PV isolation can lead to improved outcomes in AF patients treated with catheter ablation who experience recurrence and complication rates not significantly lower than with surgical ablation according to available trial data [1].

### Efficacy

In our study, surgical AF ablation was unsuccessful in 114 patients (i.e. one out of five) likely due to the lack of mapping and catheter ablation. This leads to the second consideration, the efficacy of the proposed treatment. Part of the answer to this problem is an evolution of our AF ablation strategy with the addition of a "hybrid" approach and ablation of the roof of the left atrium [8].

A strength of our study lies in the assessment of the patients’ quality of life after the procedure, which differs from previous reports on thoracoscopic ablation that mainly focused on AF recurrence. A recent meta-analysis showed a significantly higher freedom from atrial tachyarrhythmia and less need for repeat ablations after thoracoscopic ablation compared with AF catheter ablation [9].

The Atrial Fibrillation Catheter Ablation Versus Surgical Ablation Treatment (FAST) trial randomized patients who were prone to AF catheter ablation failure (i.e. failed previous ablation or left atrial dilation and hypertension) and reported common but substantially lower recurrence after thoracoscopic compared with AF catheter ablation (56% vs. 87%) at long-term follow-up [10]. Both isolated thoracoscopic ablation and hybrid ablation were found to be significantly effective in reducing the risk for AF recurrence, but no studies evaluated procedural results in terms of patient-reported outcomes.

At present, guideline recommendations suggest that, after careful assessment of the risk–benefit ratio of surgical vs. catheter ablation, it seems reasonable to consider thoracoscopic surgery preferentially in patients with previous failed catheter ablation or at high risk of catheter ablation failure [1]. In addition, thoracoscopic surgery may also be considered as first-line therapy for patients who remain highly symptomatic despite optimal medical therapy, with a class IIb recommendation due to lack of data in patients treated using this approach [1].

Therefore, up today, cardiac surgery continues to play a role within the AF heart team providing patients with a treatment option that, according to the results from randomized controlled trials, is more effective than catheter ablation in achieving rhythm control [1]. It seems that some definite conclusions set by current guidelines are often misread.

### Perspective

Given the not negligible proportion of patients experiencing failed ablations with subsequent poorer long-term clinical outcomes and quality of life, it can be speculated that hybrid surgical-catheter ablation procedures combining a minimally invasive epicardial ablation with no sternotomy and cardiopulmonary bypass with a percutaneous endocardial approach may result in improved outcomes than either procedure alone [11]. However, available data are to be considered preliminary, in that they are mainly focused on the risk of recurrent AF and no comparisons have been made between the two treatment strategies using either the one-step or two-step procedure, leaving many questions unanswered.

### Originality

Earlier studies undertaken before the latest guidelines have shown that isolated surgical ablation with right or bilateral thoracoscopic approach or subxiphoid access can improve patient outcome [12, 13]. However, the originality of our study lies in the use of minithoracotomy in the largest case series reported so far, which was performed as an isolated procedure instead of adopting a hybrid approach, though this may represent a limitation of our study. However, our preliminary experience with an hybrid approach was still published with encouraging results [14].

### Study limitations

Several limitations should be acknowledged in our study. Previous findings from our group demonstrated that increased left atrial dimension and high homocysteine levels during follow-up were predictors of AF recurrence after surgical ablation [15]. In this study, however, we could not evaluate these factors as these data were not available for all patients. For the same reason, no assessment could be made of the possible effects of antiarrhythmic and anticoagulant therapy during follow-up. Furthermore, future studies will have to compare our strategy with that of a left-sided thoracoscopy; as well as, to understand if the associated closure of the left auricle can be associated with a further improvement of the cerebro-embolic outcome at the follow-up. Our study has also limitations inherent to its design. The new approach based on patient-reported outcomes led us to measure NYHA class and EHRA score at follow-up in order to assess patients’ quality of life. However, the lack of pre-procedural data for comparison did not allow us to use a particular quality of life scale (e.g. SF-36) in all patients. Similarly, specific echocardiographic measurements that may be associated with recurrent AF (e.g. left atrial dimensions) were not available for all patients and it was decided not to include these data.

## Conclusions

In conclusion, we believe that both isolated surgical or catheter ablation for AF are destined to provide unsatisfactory results but, at present, only few data are available for the hybrid approach. It would be interesting to understand why the high number of catheter ablation procedures is not counterbalanced by a proportionate number of hybrid procedures.

The results of our study show that surgical AF ablation through a right minithoracotomy is safe and even safer than thoracoscopic or catheter ablation according to available evidence. In order to improve patients’ cardiac function and quality of life after failed AF ablation, we hypothesize that higher procedural success rates could be obtained from better management of these patients within the heart team and the adoption of a hybrid approach to achieve complete closure of the box lesion.

## Figures and Tables

**Fig. 2:**
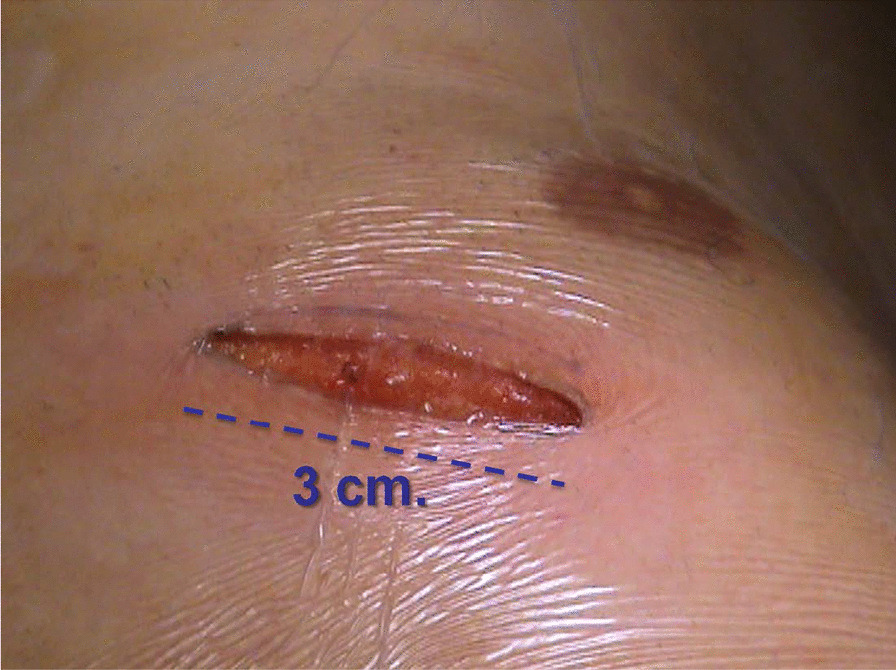
Surgical approach to atrial fibrillation ablation through minithoracotomy

**Fig. 3:**
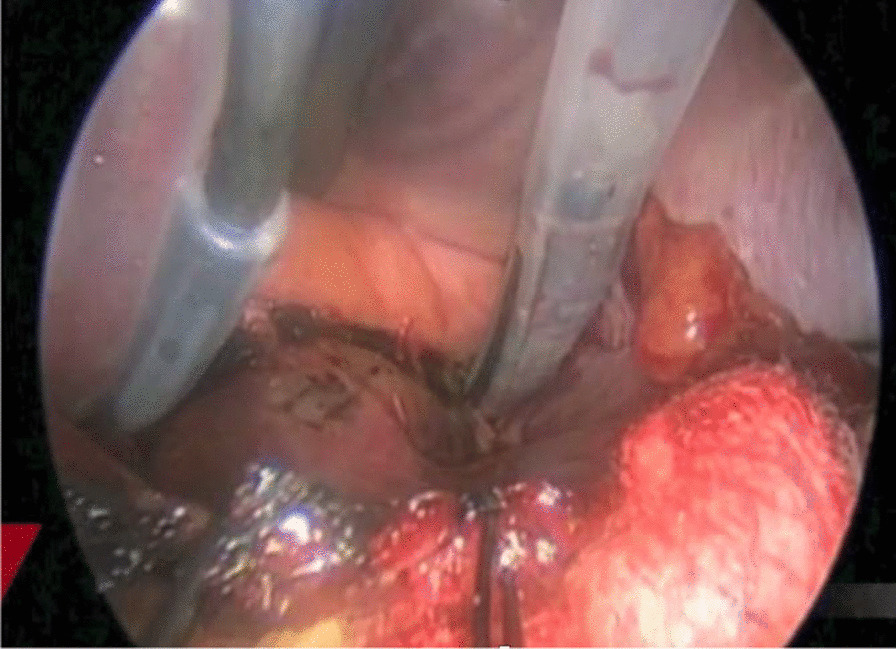
Placement of the ablation device for creating the box lesion and the visible scar (black line) post-ablation

## Data Availability

The datasets used and/or analysed during the current study are available from the corresponding author on reasonable request.

## Appendix 1

See Fig. 2.

## Appendix 2

See Fig. 3.

## Abbreviations

AF: Atrial fibrillation
EACTS: European Association of Cardio-Thoracic Surgery
ECG: Electrocardiographic
NYHA: New York Heart Association
EHRA: European Heart Rhythm Association
ESC: European Society of Cardiology
GVM: Gruppo Villa Maria
PV: Pulmonary vein
